# Confined photo-release of nitric oxide with simultaneous two-photon fluorescence tracking in a cellular system

**DOI:** 10.1038/s41598-018-27939-4

**Published:** 2018-06-27

**Authors:** Hanna Thomsen, Nino Marino, Sabrina Conoci, Salvatore Sortino, Marica B. Ericson

**Affiliations:** 1 University of Gothenburg, Department of Chemistry and Molecular Biology, Biomedical Photonics Group, Gothenburg, 412 96 Sweden; 2 University of Catania, Department of Drug Sciences, Laboratory of Photochemistry, Catania, 95125 Italy; 3 STMicroelectronics, Catania, 95121 Italy

## Abstract

Nitric oxide (NO) is a key signaling molecule in biological systems. New tools are required to therapeutically modulate NO levels with confined precision. This study explores the photoactivatable properties of an NO releasing compound (CPA), based on cupferron O-alkylated with an anthracene derivative. Upon light stimulation, CPA uncages two species: cupferron, which liberates NO, and an anthrylmethyl carbocation, which evolves into a fluorescent reporter. Proof-of-principle is demonstrated using one- and two-photon excitation (1PE and 2PE) in a cellular system (A431 cells). It was found that 1PE induces cell toxicity, while 2PE does not. Since 1PE using UV light is more likely to generate cellular photodamage, the cell toxicity observed using 1PE is most likely a combinatory effect of NO release and other UV-induced damage, which should be subject to further investigation. On the other hand, absence of phototoxicity using 2PE suggests that NO alone is not cytotoxic. This leads to the conclusion that the concept of 2PE photorelease of NO from CPA enable opportunities for biological studies of NO signaling with confined precision of NO release with minimal cytotoxicity.

## Introduction

Nitric oxide (NO) is a small diatomic molecule that plays a pivotal role in several biological and physiological processes^1–6^. The discoveries describing the signaling functions of NO in the cardiovascular system were awarded the Nobel Prize in 1998^7^. Today it is well accepted that the intricate functions of NO range from being a signaling molecule in endothelial and neural cells, to being a killer compound as utilized by immunologic cells^6,8–12^. Because of the complex role of NO in several biological functions, it is desirable to develop tools enabling controlled NO release, recently highlighted in a review by Bogdan^12^. A strategy would be to stimulate endogenous cellular production of NO, hampered by the complexity of controlling the NO generating pathways. Light, in combination with a suitable NO photoreleasing compound, can provide a powerful and minimally invasive “microsyringe” for the injection of NO into biological systems with spatiotemporal accuracy. Various NO-photodonors have been proposed^13–17^, however, further exploration is required, particularly in context of biological applicability and cell toxicity.

**Figure 1:**
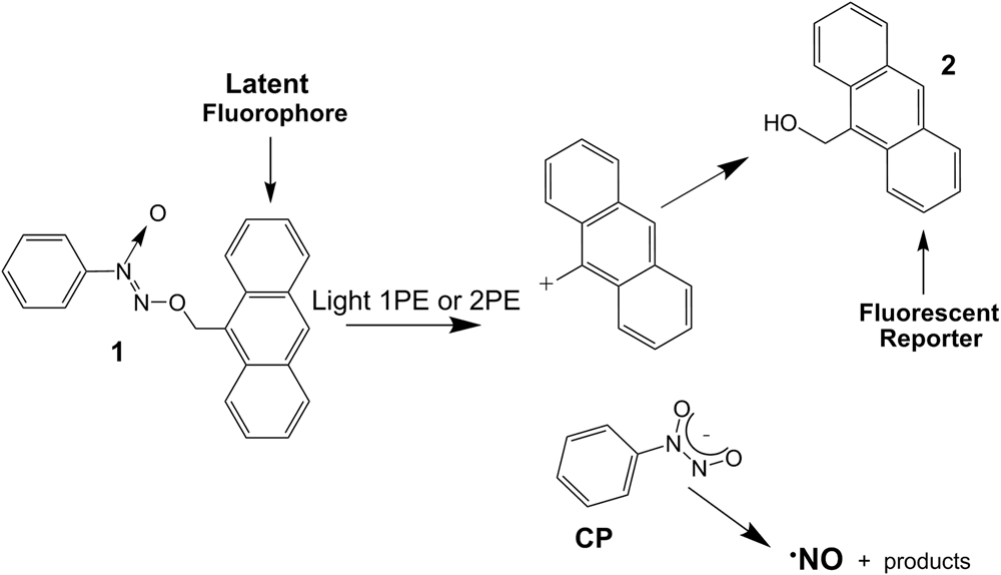
Cupferron (CP) *O*-alkylated with anthracene (CPA, compound 1) which, upon light activation, one-photon (1PE) or two-photon (2PE) excitation, forms anthrylmethyl carbocation and CP. Due to charge transfer the fluorescence of CPA (1) is quenched, while after light activation the fluorescent co-product (2) is formed, and CP spontaneously decompose to release nitric oxide (NO).

The quantification of the NO delivery in the biological system is another important issue to be faced. One possibility to address this task is to use a fluorescent reporter. This strategy relies on the simultaneous photo-release of the desired caged bioactive species and a fluorescent component from the same non-fluorescent caged precursor^18–20^. In such a way, the uncaging process can be quantified by “non-invasively” monitoring the fluorescence emission of the reporter. In addition, the spatio- temporal distribution can be observed in real time by fluorescence microscopy. Cupferrons (CP) are spontaneous NO donors at room temperature, while their *O*-alkylated counterparts have been found stable^21^. Inspired by initial work by Wang and co-workers^21^, we have previously shown^22^ that CP *O*-alkylated with anthracene (CPA, compound **1**, Fig. 1) is stable at room temperature. Excitation of the anthracene moiety at 380 nm leads to uncaging of CP, which spontaneously liberates NO. In addition, an antrylmethyl carbocation is simultaneously formed as key intermediate in the photodecomposition. The precursor for this photo-reactivity pathway was demonstrated to be a charge transfer state which is also responsible for the strong fluorescence quenching of the anthracene moiety^22^.

In the present paper, we explore the photoactivatable properties of compound **1** by using one-photon excitation (1PE) and two-photon excitation (2PE), put in a biological context. In particular we demonstrate that i) photoexcitation of **1** can be performed in a cellular system under biological conditions; ii) the formation of fluorescent co-product **2** (Fig. 1) can be traced in a biological system functioning as an optical reporter for NO release; and iii) that NO induced cell mortality appears to be dependent on the photoexcitation modality (*i*.*e*. 1PE versus 2PE).

## Results and Discussion

### Photophysical characterization

**Figure 2:**
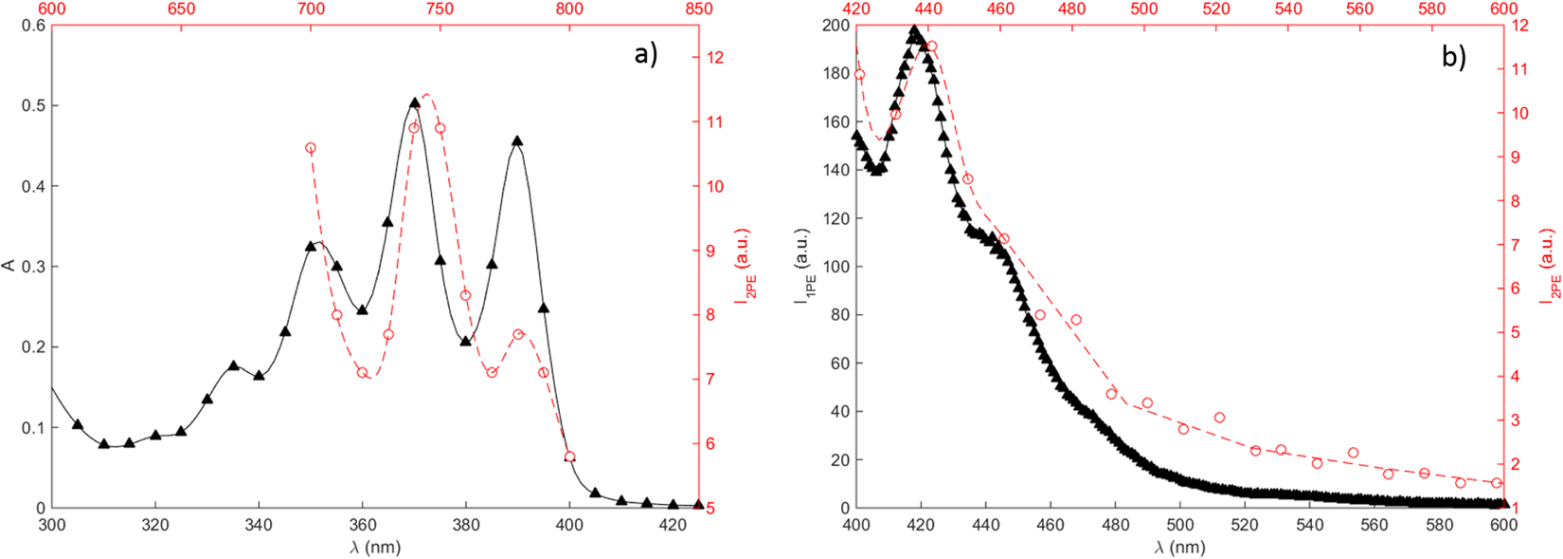
Spectral characterization of 2.5% CPA in FBS-free media. (**a**) Absorption (black triangles, solid line) and two-photon excitation spectrum (red open circles, dashed line), and (**b**) emission spectrum (black triangles, solid line) using 370 nm excitation and two-photon emission spectrum (red open circles, dashed line) using 745 nm excitation. The two-photon excitation spectrum in (**a**) was acquired in the multiphoton microscope using the excitation scan macro, sequentially changing the excitation wavelength in the range 700–800 nm (10 nm steps), recording the emission between 421–533 nm.

A spectroscopic investigation of CPA in FBS-free media was performed and the data presented in Fig. 2. As shown by the figure, the absorption spectra in this spectral range exhibit three absorption peaks, corresponding to the peaks observed for anthracene in methanol solution^22^. Also shown in the figure are the emission spectra acquired for 1PE and 2PE. The spectra confirms that the same spectral features can be observed using both excitation routes. This means that the same energetic transitions occurring in the one-photon case can be accessed by adopting 2PE in this wavelength range. Furthermore, the absorption and emission spectra were not significantly affected by the presence of cell media, which is of importance in cell studies.

It should be noted that despite the fact that the fluorescence of CPA is significantly quenched by a factor of 100, due to charge transfer between the anthracene and CP moieties, the fluorescence emission was sufficient in order to acquire the spectra before subsequent photo-activation. In addition, the actual two-photon excitation crossection for CPA cannot easily be determined because of the strong quenching of emission due to coupling to cupferron, and thereby dependence of fluorescence quantum yield upon irradiation; however, the two-photon absorption crossection (δ_TPA_) for anthracene in benzene solution has earlier been reported to be 1–3 × 10^−53^ cm^4^ s/photon in this energy range^23^, which can be used as reference.

**Figure 3:**
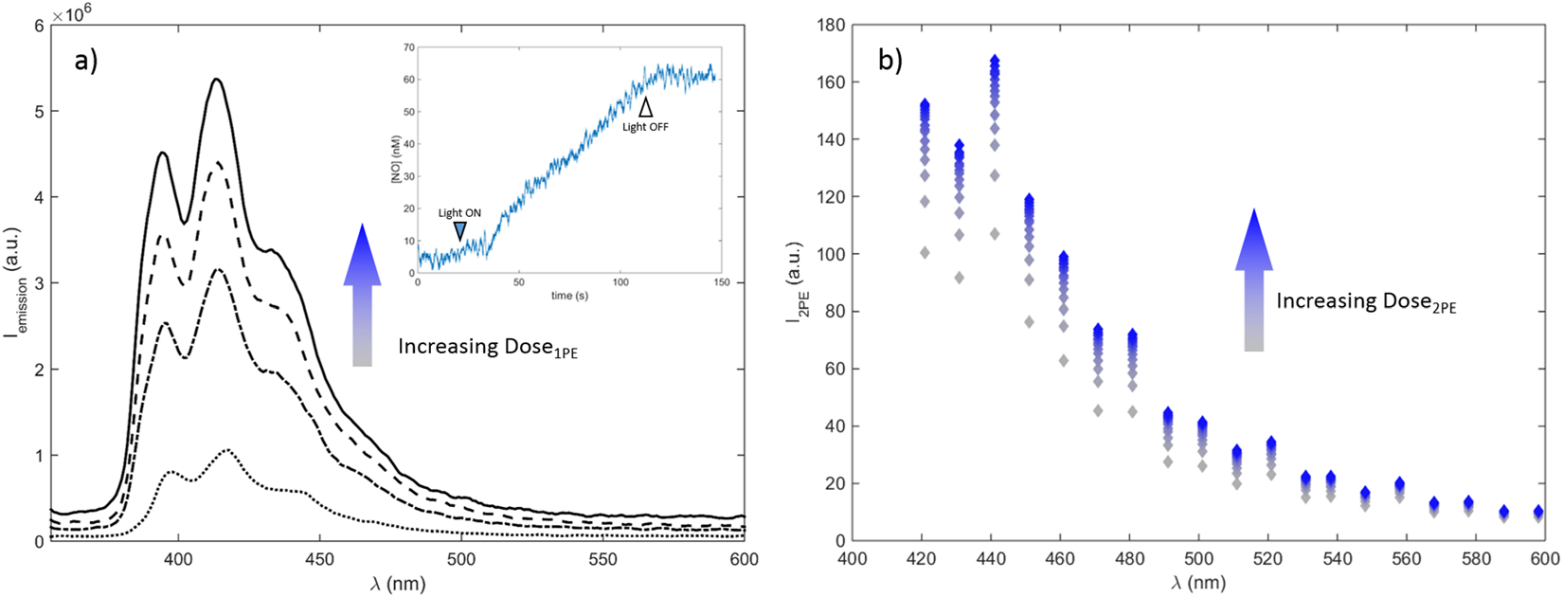
Evolution of the fluorescence emission from CPA upon one photon (**a**) and two photon irradiation (**b**). (**a**) Sequential one photon spectra (λ_exc_ = 335 nm) observed for CPA in H_2_O:MeOH (1:1) solution at increasing light dose of 386 nm at 0, 4, 8 and 18 min of irradiation from bottom to top. Insert shows the release of NO upon 390 nm irradiation; (**b**) Two-photon activation of 2.5% CPA in FBS-free media acquired in the MPM set up using sequential laser scanning with 745 nm excitation in the MPM set up.

Figure 3 shows the evolution of the fluorescence emission spectra observed at different irradiation times of CPA under 1PE and 2PE conditions. The figure indicates that photodecomposition leads to increase of the characteristic emission arising from anthracene, confirming formation of a fluorescent anthracene product. The correlation between elevated fluorescence signal and generation of NO was confirmed by the direct detection of NO through amperometric method upon 1PE of a H_2_O:MeOH solution of CPA **1** (Fig. 3a insert) in agreement with previous results^22^, implying that the system could be applied as an optical reporter system for NO release. The figure also demonstrates an increase in 2PE induced fluorescence after sequential scanning of CPA in FBS free cell media using fs-pulsed near infrared light in the multiphoton microscope. This confirms the ability of using 2PE to photo-activate CPA, in a similar manner as for 1PE photo-activation. Thus the results demonstrate the potential for using the CPA as a fluorescence reporter related to photoactivatable release of NO using also 2PE in a biological context, where direct electrochemical detection of NO under physiological conditions is analytically complex^24^.

The formed anthracene product is expected to be rather stable due to reaction of the anthrylmethyl carbocation and H_2_O. This is further confirmed by the ESI-MS analysis of the crude reaction mixture carried out immediately after the photolysis experiments (ca. 20% transformation). A main peak with a [M + 1]^+^ value of 209.2, according to the structure of compound **2** (Fig. 1) was revealed, in addition to that of the remaining starting compound.

### Photo-activation of CPA in mammalian cells

**Figure 4:**
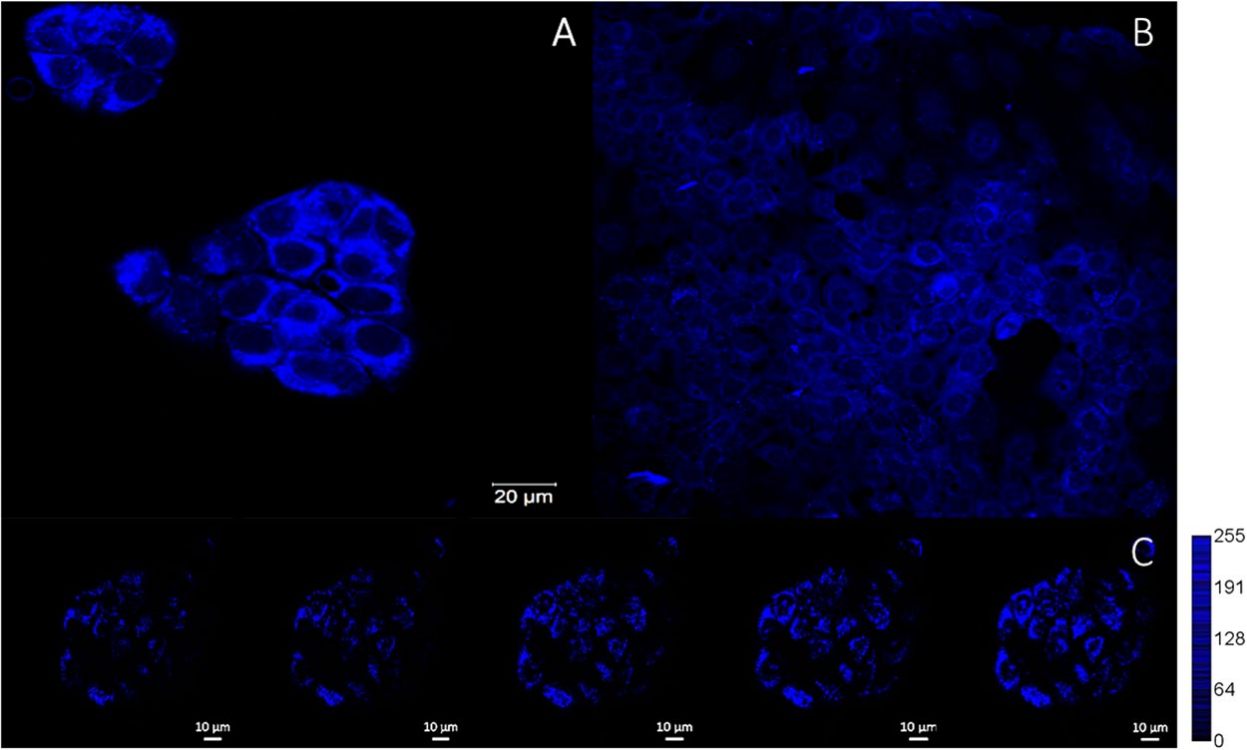
(**A**) 2PE microscopy image (λ_ex_ = 745 nm), and (**B**) confocal image (λ_ex_ = 405 nm) of A431 cells incubated with 20% CPA in FBS-free media for 4 hours. Cells were fixed only for confocal imaging. (**C**) A431 cells incubated with 5% CPA for 4 hours, subject to 2PE photoactivation (λ_ex_ = 745 nm), with increasing light doses (0.9 J, 1.7 J, 2.6 J, and 4.4 J) using a laser power of 19 mW at the sample.

The biological applicability of CPA as a photoactivatable NO releasing compound was here explored on mammalian cells (A431). To study the cellular localization of CPA, 2PE and confocal laser scanning microscopy were performed, as shown by Fig. 4. The microscopy images demonstrate that the fluorescence is primarily restricted to the cell cytoplasm. This implies that the compound is taken up by the cells, but is unlikely to enter the cell nuclei. In addition to imaging, the cells were also subject to 2PE photo-activation (Fig. 4C and Supplemental Video S1). As shown by the figure, an increase in fluorescence intensity is observed following each subsequent laser scan, in agreement with results for 2PE photo-activation in solution (Fig. 3), demonstrating proof-of-principle that 2PE can be used to stimulate activation of CPA in a biological environment. This implies that CPA can be triggered to release NO in a biological system, using the confinement potential of 2PE.

**Figure 5:**
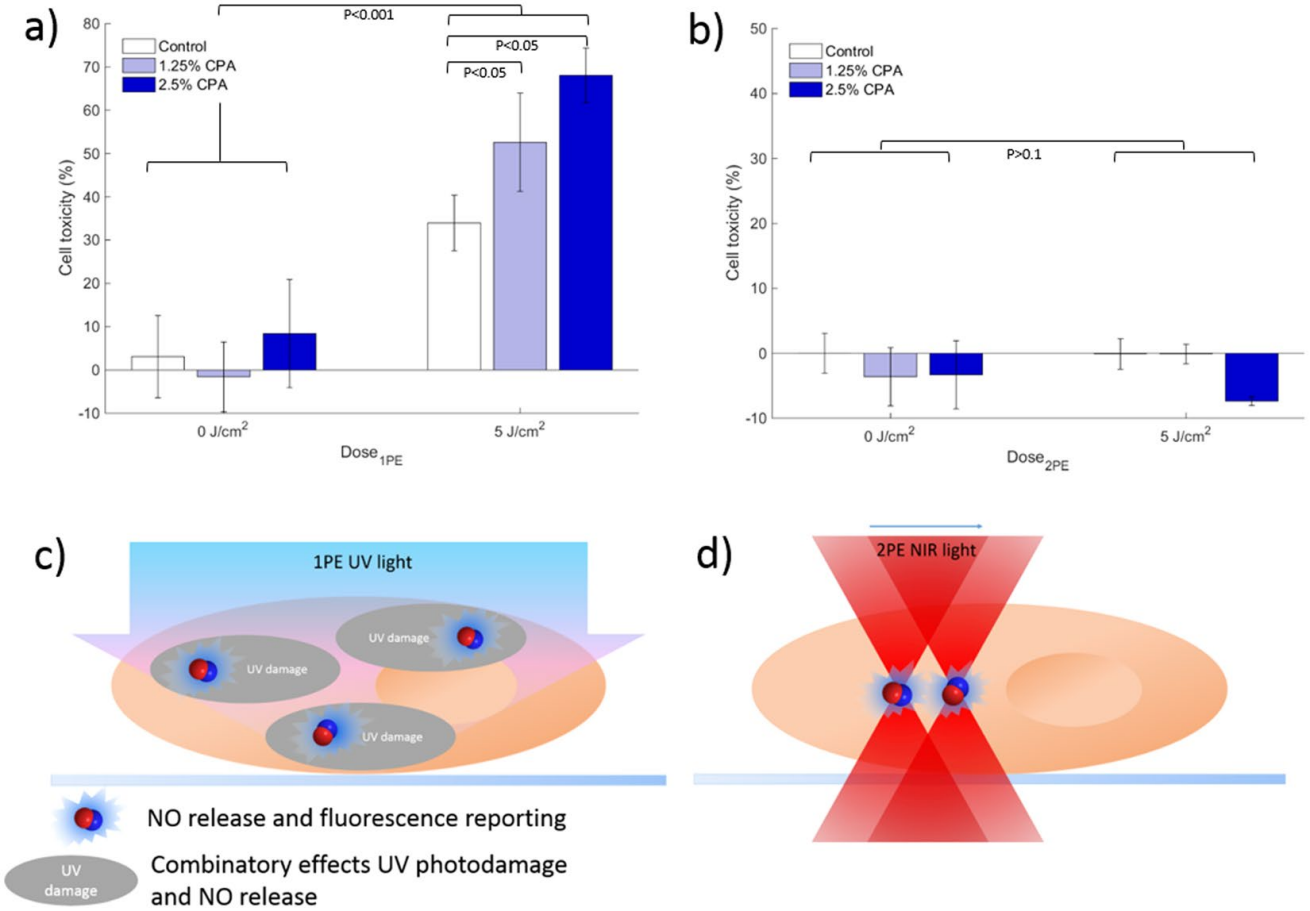
Cell toxicity experiments after photoactivation of CPA in A431 cells; (**a**) one-photon (1PE) activation using UV-lamp at light doses of 0 or 5 J/cm^2^; and (**b**) two-photon (2PE) activation using mosaic scanning and corresponding light doses of 0 and 5 J/cm^2^. Both 1PE and 2PE (**a**,**b**) performed using CPA concentration of 1.25% (grey bars) and 2.5% (open bars) compared to control with cell media only (closed bars). (**c**) and (**d**) schematic illustration of difference between 1PE and 2PE illumination conditions; For cell toxicity, statistical analysis performed from replicates of n = 6 (a and e) and n = 3 (**b**). Error bars represent standard error of mean of replicates.

The cell toxicity after irradiation was investigated, as the generation of NO was expected to have cytotoxic effects. Figure 5 shows the resulting cell toxicity of A431 cells incubated with CPA at different concentrations (0%, 1.25%, and 2.5%) after different light dosages of 1PE (Fig. 5a) and 2PE (Fig. 5b). As shown by the figure, a statistically significant increase in cell toxicity (P < 0.001) was observed with increasing 1PE irradiation dosage compared to dark control at both concentrations tested. Also seen from the figure is a dose-response effect upon UV irradiation with increasing concentration of CPA. A slightly elevated dark toxicity was observed at the highest concentration (*i*.*e*. 2.5%), but this was not found to inflict on the interpretation of dose-response effect, supporting the interpretation that photodecomposition of the compound elevates the cytotoxicity due to release of cytotoxic species.

On the contrary, when using 2PE, a lack of photo-activated cell toxicity was observed (Fig. 5b). Because of the confinement of 2PE using an MPM set up, the experiment was designed so that the 1PE and 2PE conditions should theoretically match (See Supplemental theoretical analysis). Since the 2PE excitation volume is about 1 fL, the cell culturing plates were scanned using a ‘mosaic’ pattern covering approximately 80% of the total surface area. Each well was irradiated for approximately 100 seconds corresponding to a total deposited 5 J/cm^2^. As shown by the figure, lack of cell toxicity in contradiction to the experiments performed using 1PE and UV-light was observed. This lack of cell toxicity was unexpected, but cannot simply be attributed to confined excitation, since 2PE irradiation protocol was set up to irradiate a large region of each culture well. Instead, other mechanisms should be considered.

UV irradiation is known to spark extensive cytotoxic effects in biological systems^25,26^. Thus, the observed dose-dependent cell mortality observed using 1PE for photodecomposition of CPA is most likely associated with combinatory effects of UV induced cell damage and release of NO. An earlier proposed mechanism for cytotoxic effects of NO is through generation of peroxynitrite by the reaction between NO and superoxide^6^. A scavenging experiment was performed to test this hypothesis (Supplementary Fig. S3), giving an indication that formation of peroxynitrite might play a role for the dose-dependent cytotoxic effect observed for 1PE photoactivation of CPA; however, other combinatory factors such as UV induced DNA damage and photoionization effects of anthracene cannot be ruled out. On the other hand, the lack of cell toxicity following 2PE induced photoactivation of CPA supports that photodecomposition of CPA alone is not cytotoxic. Because of the confinement of the 2PE excitation process as illustrated by Fig. 5c, in combination with minimal photoinduced cytotoxicity of NIR excitation, the cytotoxic effects of NO release seem to be minimized. This means that CPA in combination with 2PE photo-activation accounts for an interesting system for triggering and monitoring NO release with confined precision in biological environment with minimum cell toxicity. This opens up novel areas for in depth mechanistic studies of NO signaling, known to be a key player in a multitude of biological functions, *e*.*g*., innate and adaptive immunology^12^, neural transmission^27^, and endothelium response^28^, and is of particular importance for any studies translating to more complex systems than cell monolayers.

## Conclusion

In this study, we explore the potential of obtaining light stimulated NO release using a dual-function photocaged compound, which upon light activation becomes fluorescent simultaneously as NO is released. The specific compound studied was obtained by conjugation of *O*-alkylated cupferron with anthracene, to form thermostable caged NO releasing CPA. The experiments show that photo-induced decomposition of CPA can be obtained using near infrared (NIR) light, utilizing the process of 2PE, which makes it a suitable candidate for the study of confined NO release in biological samples. Because of the inherent properties of the 2PE processes, spatial confinement of NO release can be restricted ultimately to single cell studies, and NO release generated in a sub-fL volume, opening up new possibilities for mechanistic studies of NO signaling.

When CPA is activated using UV-light and 1PE, the compound demonstrates cytotoxic properties. Both confocal and two-photon fluorescence microscopy indicate cytosolic localization of CPA, and limited access to cell nuclei. Interestingly, the compound was only found to be cytotoxic when the light stimulation was obtained with 1PE and not 2PE. This could most likely be attributed to that combinatory effect of NO release and 1PE induced UV damage. Since NIR is less prone to induce photoinduced cytotoxicity, this opens up novel possibilities in exploring photoactivatable NO releasing compounds with confined 2PE for biological studies of NO signaling, with minimal cytotoxicity. In combination with low inherent phototoxicity of the NO releasing compound, this serves as a novel system for future studies of NO regulatory processes in biological tissue, subject to further investigations.

## Methods

### Synthesis

Cupferron-antracene (CPA) was synthesized and characterized as previously described^22^ using the procedure introduced by Hou *et al*.^21^. Briefly, 9-chloromethyl anthracene were injected through a septum to a solution of cupferron in DMF cooled at 0 °C and stirred overnight. The reaction mixture was diluted with water, extracted with CH_2_Cl_2_ and products purified after solvent evaporation by silica gel chromatography using cyclohexane/ethyl acetate 80:20. (Z)-2-(9-Anthrylmethoxy)-1-phenyldiazene 1-oxide: Elemental analysis calcd (%) for C21 H16N2O2: C 76.81, H 4.91, N 8.53; found: C 76.05, H 4.82, N 8.21; ESI-MS: m/z (%): 351.1 (100) [M + Na] + ; 1H NMR (CDCl3, 500 MHz): d = 8.55 (s, 1 H), 8.50 (d, 2 H, J = 8.5 Hz), 8.04 (d, 2 H, J = 8 Hz), 7.86 (dd, 2 H, J1 = 8.4, J2 = 7.5 Hz), 7.61 (dd, 2 H, J1 = 8, J2 = 7.4 Hz), 7.50 (m, 2 H), 7.45 (m, 1 H), 7.40 (m, 2 H), 6.45 ppm (s, 2 H).

### One-photon spectroscopy

UV/vis absorption spectra were recorded using a Cary UV-Vis spectrophotometer (Agilent Technologies AB, Kista, Sweden). Fluorescence spectra were acquired by fluorescence spectrophotometer (Cary Eclipse, Varian AB, Bromma, Sweden) and a Fluorolog 2 mod F-111 spectrofluorimeter. Spectral measurements were performed with a 2.5% dilution of stock solution 25 mg/mL CPA in 100% DMSO in FBS-free MEM media using quartz cuvette (1 cm path length, 3 mL capacity). Absorption and emission spectra measurements were corrected for solvent background.

NO release after one-photon irradiation was measured by amperometric technique as earlier described^22^. In brief a ISO-NO meter (World Precision Instrument) was used by positioning the electrode outside of the light path in thermostated quartz cell (1 cm path length, 3 ml capacity) containing a solution of CPA in H2O:MeOH (1:1). Irradiation was performed 390 nm of the Fluorolog-2 (see above) as light source.

### Two-photon laser scanning microscopy

2PE was obtained using two different systems. For imaging, a commercial multiphoton microscope (LSM 710 NLO microscope, Carl Zeiss, Jena, Germany) was used. The system is equipped with mode-locked femtosecond pulsed Mai Tai DeepSee laser (SpectraPhysics, Stahnsdorf, Germany) operating at 80 MHz, tunable in the wavelength region 700–1100 nm. A Plan-Apochromat 20X water immersion objective (NA 1.0) was used in the experiments. Fluorescence was registered with descanned (internal) detectors with a fully opened pinhole. Emission spectra were acquired by using the spectral detector on the system. After calibration it was found that emission spectra using the microscope was 20 nm shifted compared to emission acquired by the spectrophotometer which was corrected for in the analysis. Excitation scan was performed using a built-in excitation scan macro. For imaging, the excitation wavelength was set to 745 nm, and emission recorded in one channel (421–533 nm). Laser power at the sample was approximately 19 mW. Images were acquired with a pixel dwell time of 1.58 μs. The image frame size was 1024 × 1024 pixels for all images presented. Image processing was performed with ZEN (Carl Zeiss, Jena, Germany) and ImageJ (U.S. National Institutes of Health, Bethesda, Maryland).

For toxicity experiments using 2PE, an experimental MPM platform was used. The system is an inverted Zeiss axiovert 135 TV with a Ti:Sapphire mode locked laser (Tsunami, Spectra physics, Mountain View CA, USA) was used as the excitation source of the setup. The laser is tunable in the wavelength range 700–1050 nm, and provides a repetition rate of 80 Hz with pulse duration of ~100 fs. A pump power of 6 W gives a laser beam of 800 mW as the beam exits the laser cavity.

### Cell culture

Minimum essential media (MEM), phosphate buffered saline (1 X PBS pH 7.4), glutamine, and non-essential amino acids were obtained from Thermo Fischer Scientific, Gothenburg, Sweden. Human squamous carcinoma cells (A431, HPA cultures, Salisbury, UK) were cultured at 2 × 10^5^ cell per mL in full-growth MEM media supplemented with 10% fetal bovine serum (FBS, EU approved South American origin, Thermo Fischer Scientific, Gothenburg, Sweden), 5% glutamine, and 5% non-essential amino acids in 37 degrees Celsius at 5% CO_2_. Prior to experiments, cells were seeded in 96-well plates (10 000 per well) or tissue culture treated petri dishes (∅ = 3 cm, 60 000 cells per mL, Ibidi, LRI Instrumenets, Lund, Sweden) for one-photon and two-photon experiments respectively, and incubated for 24 hours. Cell culturing was performed in accordance with biomedical guidelines^29^ and local regulations.

CPA-media solutions (0.75%, 1.25%, 2.5%, 5%, and 10%) were prepared by adding CPA from a stock solution (2.5 mg/mL) in 100% DMSO (Sigma-Aldrich, Stockholm, Sweden) to FBS-free cell media. Four hours prior to irradiation, cell media was replaced with 100 μL CPA-media solution ranging from 830 μM (10% stock CPA solution in media) to 62 μM (0.75% stock CPA solution in media). The maximum total concentration of DMSO in the cell experiments were kept below 5%, to ensure that cell viability was not affected (See supplementary data).

Prior to confocal imaging, cells were fixed using the following procedure: A 4% formaldehyde solution was prepared in PBS. Cells were washed carefully with PBS and formaldehyde solution was added to the cells for 20 minutes at room temperature. Cells were then washed carefully twice with PBS and 2 mL PBS was added to the petri dish for imaging.

### Cell toxicity assay

To assess cell toxicity the AlamarBlue cell viability protocol was used, the assay was performed following manufacturer protocol. Briefly, AlamarBlue reagent (Thermo Fisher Scientific, Stockholm, Sweden) was added to each well as 10% of the sample volume and allowed to incubate for 4 hours. Following incubation, the absorbance of AlamarBlue was measured at 570 nm and 600 nm using a SpectraMax M2 Multi-mode microplate reader (Molecular Devices, Berkshire, UK). Results were analyzed by plotting absorbance vs compound concentration as described in manufacturer protocol. Cell toxicity was further confirmed using the MTT colorimetric cell metabolic activity (supplementary data).

### 1PE photo-activation

The cells in 96 well plates, incubated with CPA at varying concentrations for 4 hours, were subject to irradiation using a UV-lamp (UV-B TL 20 W, emission range 300–350 nm. Phillips AB, Malmö, Sweden). One column of wells was supplemented with 100% full growth media (100 μL) serving as control. One-photon photoactivation experiments were performed in replicates of 6. Immediately prior to irradiation the solution was removed from the wells and cells were gently washed with PBS. Full-growth media (200 μL) was added to each well. Plates were irradiated in increments of 0, 30, and 60 seconds, corresponding to irradiation dosage 0, 5, and 10 J/cm^2^ respectively. Parallel to the irradiation experiments, an additional 96-well plate was prepared analogously but kept in the dark outside of the incubator, serving as dark control. Both plates were then allowed to recover for 24 h in the incubator before subject to toxicity assay.

### 2PE photo-activation and visualization

The cells in the petri dishes were incubated with FBS-free media containing 2.5% CPA for 4 hours, corresponding to the highest dosage of CPA producing statistically significant cell toxicity in one-photon experiments. Immediately prior to experiment, the solution was removed and cells were gently washed with PBS. Experiments were performed after adding 2 mL PBS to the petri dish and placing it on the multiphoton microscopy stage. The laser wavelength was set to 745 nm for both imaging and photoactivation. Photoactivation was performed using the photobleaching function of the microscope. The cells were scanned repeatedly using a linear scanning pattern in a chosen region of interest of 1024 × 1024 pixels, corresponding to 142 × 142 μm with 3x zoom. After each exposure, a fluorescence image of the scanned cells was recorded and saved. Cells were subjected to 15 sequential scans with increasing dosage of photoactivation from 0 to 217 s, corresponding to 0 to 4.4 J irradiation dose. Fluorescent images after each scanning event were compiled for analysis of fluorescence intensity changes. In addition, fluorescence spectra were captured for each time point using the lambda-scanning function of the microscope, generating fluorescence emission spectra in the range 421–700 nm (9.7 nm steps).

### 2PE photo-activation and cell toxicity

Cells were cultured as described previously and seeded in 96 well plates at a concentration of 20,000 cells per well. Two-photon photoactivation experiments were performed in replicates of 3. Cells were incubated with 1.25% and 2.5% CPA stock solution in FBS-free media as described previously, maintaining one column as a control sample with cells incubated only in media. Plates were fit into a 96-well plate holder on the microscope stage of the experimental MPM set up. A LabView program was created to perform automated sequential photoactivation of each sample well in the plate. Wells were irradiated using a ‘mosaic’ pattern of covering approximately 80% of the well. Each well was irradiated for approximately 100 seconds corresponding to approximately 5 J/cm^2^. Parallel to the irradiation experiments, an additional 96-well plate was prepared analogously but kept in the dark outside of the incubator, serving as dark control. Both plates were then allowed to recover for 24 h in the incubator before subject to toxicity assay as described above.

### Statistical analysis

Experiments were done in replicates of n = 6 for 1PE and n = 3 for 2PE. Statistical analysis was performed using excel data analysis toolbox student’s t-test. P-values are shown as annotations to cell toxicity graphs and standard error of mean across replicates is represented in percentage as error bars.

### Data availability

The datasets generated during and analyzed during the current study are available from the corresponding author on request.

## Electronic supplementary material

Supplementary information

Supplemental video S1
