# Supplementary material for: Confined photo-release of nitric oxide with simultaneous two-photon fluorescence tracking in a cellular system
Source: Sci Rep. 2018 Jun 27;8:9753. doi: 10.1038/s41598-018-27939-4 (PMC6021447; doi:10.1038/s41598-018-27939-4)
Supplement: Supplementary file 1 — Supplementary information [file 41598_2018_27939_MOESM1_ESM.pdf]

Confined photo-release of nitric oxide with simultaneous two-photon fluorescence tracking in a cellular system

Hanna Thomsen[1], Nino Marino[2], Sabrina Conoci[3], Salvatore Sortino[2]*, and Marica B. Ericson[1]*

[1] University of Gothenburg, Department of Chemistry and Molecular Biology, Biomedical Photonics Group, Gothenburg, 412 96, Sweden
[2] University of Catania, Department of Drug Sciences, Laboratory of Photochemistry, Catania, 95125, Italy
[3] STMicroelectronics, Catania, 95121, Italy
* ssortino@unict.it; marica.ericson@chem.gu.se

Supplemental information

- Supplemental data
- Supplemental theoretical analysis
- Supplemental video

Supplemental data

Cell toxicity screening of DMSO as drug diluent for A431 cells

Since the CPA compound was only found to be soluble in DMSA, an analysis on what suitable concentration range of DMSO was performed. A431 cells were seeded at 1x10^4 per well tissue culture treated 96 well plates (Nunc cell culture plates, ThermoFisher Scientific) and allowed to incubate at 37 degrees in 5% CO2 for 24 hours before experiment. Four hours prior to irradiation, full growth media was replaced with varying percentage (1%, 2.5%, 5%, 7.5%, 10%, 12.5%, 15%, and 20%) DMSO in FBS-free media with the exception of one column replaced with 100% full growth media for control. Toxicity of varying concentrations of DMSO was evaluated using the MTT viability assay (Sigma-Aldrich, Stockholm, Sweden). As shown by Supplemental Fig. S1, the cell toxicity was found to be strongly increased for DMSO concentration above 2.5%. Thus the DMSO concentration was kept at or below 2.5 % in in the experiments.

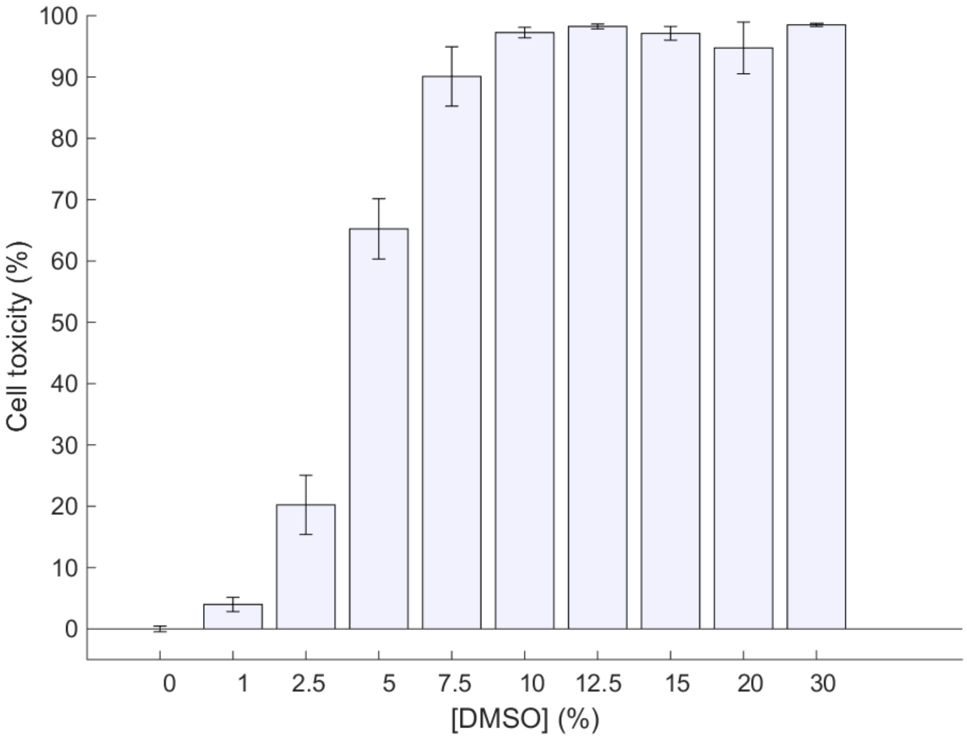

Supplemental Figure S1. Cell toxicity of A431 cells in the presence of DMSO at varying concentrations in FBS-free media (0 – 30% DMSO in media by volume). Cells were incubated with DMSO concentrations for 4 h prior to evaluation of cell viability by the MTT assay. Replicates n = 4, error bars showing standard deviation.

Cell toxicity, 1PE photoactivation using MTT assay

In addition to measuring cell toxicity following photoactivation treatment with the AlamarBlue assay; cell viability was monitored using MTT-assay using protocol as suggested by manufacturer (Sigma-Aldrich, Stockholm, Sweden). Briefly, 20 µL of MTT (5 mg/mL) in cell media was added to each well of the 96-well plates. After 4 h of incubation, media and MTT solution was replaced with 100 µL HCl 0.01 M in DMSO. Absorbance was measured at 550 nm using a SpectraMax M2 Multi-mode microplate reader (Molecular Devices, Berkshire, UK). Experiments were performed in replicates of 6. Statistical analysis was performed using excel data analysis toolbox student's t-test. P values are shown as annotations to cell viability graph and standard error of mean across 6 replicates.

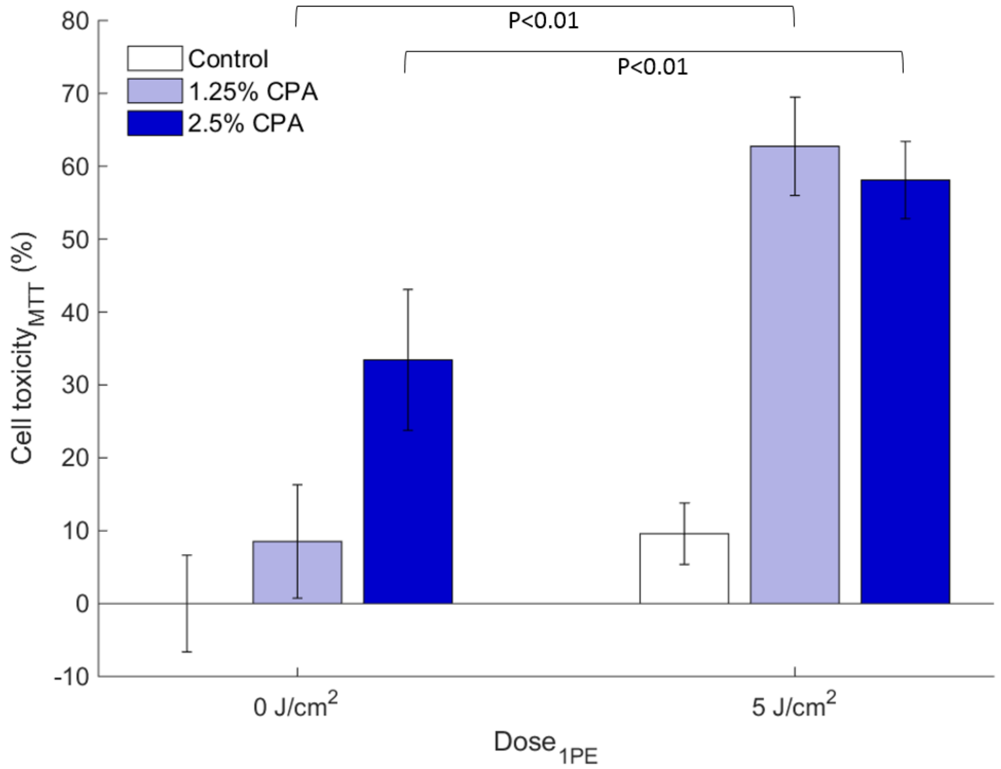

Supplemental figure S2. Cell toxicity of A431 cells using MTT assay after one-photon induced photoactivation at CPA concentration at 1.25% and 2.5% compared to control with cell media only. Photoactivation obtained using a broad spectrum UV-lamp at light doses of 0, 5 J/cm^2. Statistical analysis performed from replicates of n=6. Error bars represent standard error of mean.

Scavenging experiment

A scavenging experiment was performed, adopting a protocol by others [1]. Cells were cultured and seeded as earlier. CPA at 2.5%. was added together with either superoxide dismutase (Sigma-Aldrich, Stockholm, Sweden) as superoxide scavenger, uric acid (Sigma-Aldrich, Stockholm, Sweden) as peroxynitrite scavenger, or catalase (Merck Chemicals and Life Science, Solna, Sweden) as hydrogen peroxide scavenger, at concentrations of 100 ug/mL (PBS, pH 7.4), at the same time as addition of CPA, and incubated for 4 hours. Irradiation using UV was performed as earlier, but light dosage was adjusted to 10 J/cm^2. Immediately following irradiation, all solutions were removed and replaced with fresh media and allowed to recover overnight and cell toxicity assessed. The data is presented in Figure S3 and controls in Figure S4. No significant difference in cytotoxic effect upon irradiation compared to control cells was found when uric acid, known as peroxynitrite scavenger [1,2], was added together with the CPA. Addition of superoxide dismutase and catalase, *i.e.*, superoxide and hydrogen peroxide scavengers,respectively, showed no significant difference in cell toxicity compared to CPA only after irradiation. This experiment indicates that generation of peroxynitrite upon irradiation might be involved for causing the cell toxicity following 1PE irradiation of CPA; however to fully elucidate the mechanism further experiments should be undertaken.

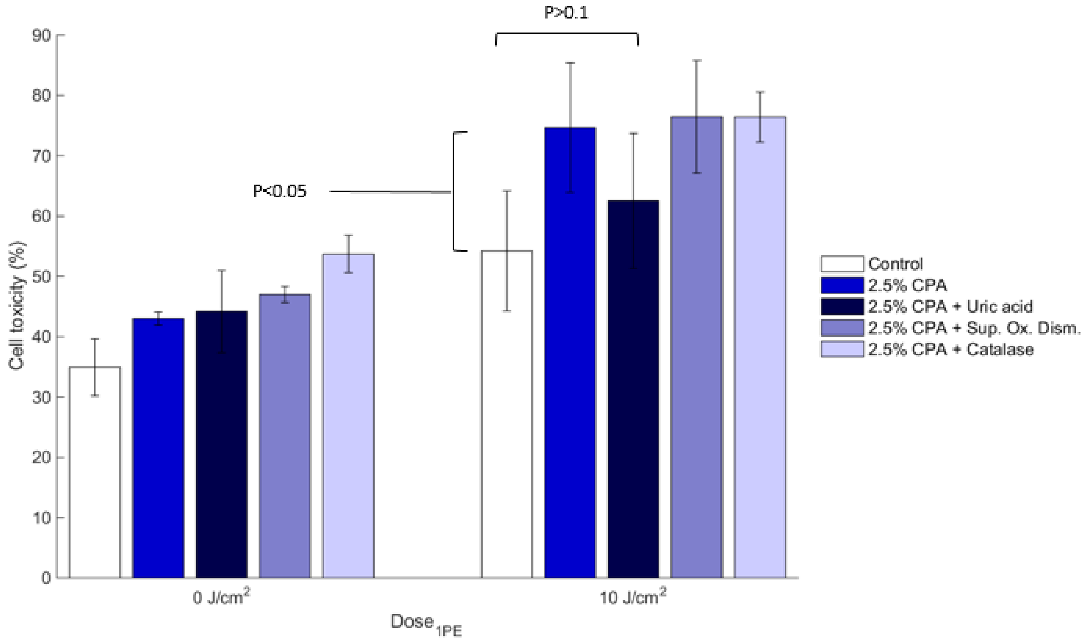

Supplemental figure S3. Scavenging experiments of A431 cells after 1PE photoactivation (2.5% CPA concentration) with addition of scavengers; uric-acid (peroxynitrite scavenger), superoxide dismutase (superoxide scavenger), and catalase (hydrogen peroxide scavenger), in concentrations of 100 µg/mL in PBS. For cell toxicity, statistical analysis performed from replicates of n=6. Error bars represent standard error of mean.

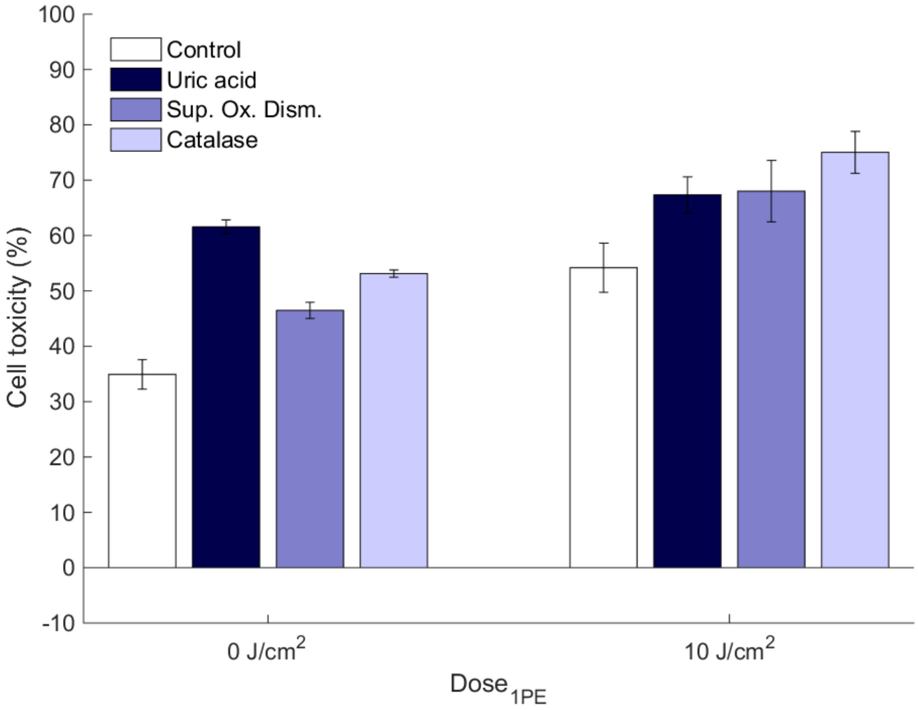

Supplemental figure S4. Control for scavenging experiment. Cell toxicity of A431 cells after 1PE photoactivation of scavengers; uric-acid (peroxynitrite scavenger), superoxide dismutase (superoxide scavenger), and catalase (hydrogen peroxide scavenger), in concentrations of 100 µg/mL in PBS. Photoactivation obtained using a broad spectrum UV-lamp at light doses of 0, 5 J/cm^2. For cell toxicity, statistical analysis performed from replicates of n=6. Error bars represent standard error of mean.

Theoretical analysis

In order to compare the likelihood for a CPA molecule undergoing photo-induced decomposition through either 1PE or 2PE using the conditions given in the study a theoretical calculation was performed. The value for molar extinction is approximately $\varepsilon = 10\ 000\ M\ cm^{-1}$ according to Vittorino et al. [3], corresponding to an absorption crossection $\sigma_{1PA} = 3.8 \times 10^{-8}\ cm^2$ (using $\sigma_{1PA} = \ln(10) \times \varepsilon / N_A$). The two photon absorption crossection is approximated to be similar to anthracene in solution $\delta_{2PA} = 1 \times 10^{-53}\ cm^4\ s^{-1}$, determined by Webman and Jortner [4].

Given the experimental conditions in the 1PE experiments using a wavelength at $\lambda_{1PE} = 370$ nm and intensity $I_{1PE} = 0.2\ W/cm^2$, yields a photon flux approximately $\Phi_{1PE} = 3 \times 10^{17}\ cm^{-2}\ s^{-1}$. For 2PE excitation, pulsed NIR light is utilized. Thus the photon flux in the 2PE case needs to be calculated for the laser pulse at the focal spot, given that the repetition rate is 80 MHz, pulse duration approximately 100 fs, lambda $\lambda_{1PE} = 750$ nm, numerical aperture of objective lens NA = 1, and laser power at sample about 20 mW, giving $\Phi_{2PE} = 1 \times 10^{30}\ cm^{-2}\ s^{-1}$.

The probability for a molecule undergoing photodecomposition and subsequent generation of NO will be proportional to the probability of undergoing excitation, which for the two different scenarios can be estimated to

$$P_{1PE} = \sigma_{1PA} \times \Phi_{1PE}$$

$$P_{2PE} = \delta_{2PA} \times (\Phi_{2PE})^2$$

stated in the units as s^{-1}. Given the numbers above the $P_{2PE} \approx 2 \times 10^{7}\ s^{-1} >> P_{1PE} \approx 1 \times 10^{3}\ s^{-1}$, which means that the probability for a specific molecule is much more likely to undergo 2PE rather than 1PE given the conditions above. So as long as the experiments are designed to cover similar total excitation volumes and total light dosages (in our study 5 J/cm^2), 2PE should in fact be much more effective to decompose CPA in order to release NO. Thus the lack of cell toxicity observed using 2PE in our experiments cannot simply be explained by lower probability of 2PE and confinement effect, but alternative explanations should be considered.

Supplemental video

Supplemental video S1 - A431 cells incubated with 5% CPA for 4 hours, subject to 2PE photoactivation (λ_{ex} = 745 nm), with increasing light doses (0.9 J, 1.7 J, 2.6 J, and 4.4 J) using laser power 19 mW at the sample. Video demonstrate the increase in fluorescence signal following light activation.

References

1. Hooper, D. C. et al. Uric acid, a natural scavenger of peroxynitrite, in experimental allergic encephalomyelitis and multiple sclerosis. *Proceedings of the National Academy of Sciences of the United States of America* **95**, 675-680 (1998).
2. Santos, C. X. C., Anjos, E. I. & Augusto, O. Uric acid oxidation by peroxynitrite: Multiple reactions, free radical formation, and amplification of lipid oxidation. *Archives of Biochemistry and Biophysics* **372**, 285-294 (1999).
3. Vittorino, E., Cicciarella, E. & Sortino, S. A "dual-function" photocage releasing nitric oxide and an anthrylmethyl cation with a single wavelength light. *Chemistry - A European Journal* **15**, 6802-6806 (2009).
4. Webman, I. & Jortner, J. Energy dependence of two-photon-absorption cross sections in anthracene. *The Journal of Chemical Physics* **50**, 2706-2716 (1969).
